# Research on memory failure prediction based on ensemble learning

**DOI:** 10.1371/journal.pone.0321954

**Published:** 2025-04-23

**Authors:** Peng Zhang, Jialiang Zhang, Yi Li

**Affiliations:** 1 School of Information Engineering, Wuhan University of Technology, Wuhan, China; 2 Alibaba Cloud, Shenzhen, China; UO: University of Okara, PAKISTAN

## Abstract

Timely prediction of memory failures is crucial for the stable operation of data centers. However, existing methods often rely on a single classifier, which can lead to inaccurate or unstable predictions. To address this, we propose a new ensemble model for predicting CE-driven memory failures, where failures occur due to a surge of correctable errors (CEs) in memory, causing server downtime. Our model combines several strong-performing classifiers, such as Random Forest, LightGBM, and XGBoost, and assigns different weights to each based on its performance. By optimizing the decision-making process, the model improves prediction accuracy. We validate the model using in-memory data from Alibaba’s data center, and the results show an accuracy of over 84%, outperforming existing single and dual-classifier models, further confirming its excellent predictive performance.

## Introduction

With the continuous progress of science and technology, cloud service has developed from a preliminary concept to a powerful and systematic technical service, gradually penetrating into all levels of modern society and becoming a core force to promote the process of social informatization [1,2]. As an important infrastructure for cloud services, data centers are particularly important in the digital era, and their stability is directly related to the efficient operation of cloud services and the smooth operation of the entire social information network. Failure or instability of data centers not only leads to the interruption of information services and business stagnation, but also may have a serious impact on the social economy.

Under complex hardware environments and diverse operating conditions, the continuous and stable operation of data center servers faces great challenges. Hardware failures are difficult to avoid completely, and among these failures, memory (DRAM)-related problems dominate, especially server failures (i.e., CE-driven failures) triggered by a large number of Correctable Errors (CE) [3–6]. Although modern ECC techniques are capable of detecting and automatically correcting certain bit errors [7–9] too many errors occurring in a short period of time can still lead to memory failures that can seriously affect server performance. Such failures may be manifested as slow page loading, delayed application response, data loss or even service interruption, which directly affects the user experience. In addition, frequent memory failures significantly increase the burden on operations and maintenance teams and weaken the overall operational efficiency of the data center.

To address this problem, researchers have begun to explore machine learning-based approaches to predict memory failures. Compared with traditional methods, machine learning models are more flexible in adapting to dynamic data and complex environments, and show excellent performance in processing large-scale logs and error information [10–14]. These methods can not only extract key features from massive data, but also finely analyze the behavioral patterns of CE errors and capture early signs of memory failures in advance, providing strong support for failure prediction [15,16]. At present, machine learning methods represented by support vector machines, random forests and gradient boosting decision trees have achieved significant results in the field of memory fault prediction [16]–[17]. Meanwhile, the rapid development of deep learning techniques brings new possibilities for memory fault prediction. For example, deep learning models such as Long Short-Term Memory Networks (LSTMs) can tap into the complex correlations between memory faults and further improve the accuracy and reliability of fault detection. These new methods provide important technical support to improve the stability of data centers and increase the efficiency of operation and maintenance [18].

Although machine learning and deep learning approaches have made significant progress in the field of memory fault prediction, existing methods still face many challenges, especially when dealing with complex and dynamically changing environments. Most of the current research relies on standalone classifiers, but these approaches suffer from the following key issues:

First, a single classifier exhibits significant limitations in dealing with data imbalance and noise. Due to the uneven distribution of training data or noise interference, a single model is prone to overfitting the training data and thus has insufficient generalization ability on unseen samples, resulting in less reliable prediction results. Especially in memory fault prediction, the dramatic fluctuation of CE error in a short period of time makes this problem more prominent. Second, although deep learning has advantages in capturing complex patterns and potential associations, its performance is highly dependent on large-scale labeled data. In the field of memory fault prediction, labeled samples are often scarce to meet the training requirements of deep learning models, resulting in their inability to realize their full potential. In addition, existing methods mostly use simple statistical features and fail to comprehensively explore the complex interactions in memory faults, thus limiting the adaptability and expressiveness of the models.In addition, existing methods fail to fully utilize the diversity among classifiers. Despite the fact that different classifiers have their own advantages in specific tasks, most methods only use a simple voting mechanism or a fixed-weight averaging strategy for fusion. This static fusion approach ignores the performance differences of classifiers in different scenarios, which restricts the improvement of overall prediction accuracy and robustness. Meanwhile, the high computational complexity of deep learning models also imposes constraints on their application in real-world deployments.

To address the above issues and fill the research gap in this area, this paper proposes an integrated model based on adaptive threshold collaborative decision making, which has significant advantages over traditional methods. The method optimizes the overall performance by dynamically adjusting the weights of each classifier in the final decision, giving full play to the advantages of different models in specific tasks. Specifically, the model introduces an adaptive threshold mechanism that dynamically adjusts the weights of the classifiers based on their historical performance, enabling the model to better adapt to the complex and changing environment of data centers (e.g., memory failure prediction). This collaborative decision-making approach significantly improves the accuracy and robustness of prediction, while effectively mitigating the problems of overfitting, insufficient samples, and unstable prediction results.

The innovative approach proposed in this paper not only solves the problems of underutilization of classifier diversity and lack of dynamic adaptability in existing research, but also provides new ideas for achieving efficient and stable performance in memory fault prediction tasks. This approach shows that fully utilizing the synergistic advantages of multiple classifiers is the key to improving model performance in complex dynamic environments, and also provides a useful reference for the improvement of machine learning and deep learning methods in memory fault prediction.The main contributions of this paper include:

i) Introducing an integrated classifier model based on adaptive threshold collaborative decision making, which integrates Random Forest, LightGBM, and XGBoost, which are the classifiers that perform well in memory CE-driven fault prediction, and assigns a corresponding decision influence factor to each base classifier based on their respective individual performances in the field of memory fault prediction, to make a collaborative decision. The performance of the model is maximized by introducing an adaptive threshold adjustment mechanism to find the optimal decision threshold at the final decision of the model. By using the grid search method to find the optimal decision influencing factors, an integrated model with optimal performance is formed.
ii) In terms of performance evaluation, this paper compares the proposed integrated model with an independent classifier and a model in which two classifications make collaborative decisions based on several key performance metrics, such as precision rate, recall rate, and F1 score, as well as other methods in the field, and the results show that the proposed integrated model based on adaptive threshold collaborative decision-making exhibits higher accuracy and superior classification performance.
iii) An in-depth analysis of the advantages of the integrated model over stand-alone classifiers and combinations of two classifiers is presented, with special emphasis on its significant results in improving, among other things, the accuracy of memory fault prediction. This detailed analysis provides useful references and insights for future research in this area.

## Related work

In recent years, the field of server fault prediction has attracted widespread attention, especially in the field of memory fault research, where machine learning methods have been increasingly applied. However, memory failure prediction faces many challenges such as high data complexity, diverse error patterns, and insufficient prediction accuracy. Current research focuses on several dimensions such as log analysis, hardware characterization, and load impact, but each has its own limitations. The following is a review of several related studies that demonstrate the current state of development of various methods and techniques in the field of memory fault prediction.

In the area of log and sensor data analysis, Giurgi et al [19] pioneered the first predictive model in the field of DRAM fault prediction. The model is based on a random forest classifier with a 4-step highly optimized sliding window, an innovative application of change point detection to screen sensor metrics with unusual trend changes prior to failures, and is fully trained and validated on log and sensor information from IBM machines. Although the model achieves 96% accuracy, it relies only on short-term temporal features, which makes it difficult to capture long-term dependent patterns of error occurrences, which may lead to unstable predictions in practical applications. Fu.Y et al [20] utilized the first publicly available DRAM fault prediction dataset released in the PAKDD 2021 AIOps competition, and used the system kernel logs and MCA logs data to leverage domain knowledge to construct a set of handcrafted features for extracting hidden useful information from raw categorized values. Their approach treats the DRAM fault detection problem as a multi-class classification task and employs a state-of-the-art XGBoost classifier, but the approach overly relies on a single log source and ignores the significant impact of the hardware itself on DRAM errors. To address this problem, Z. Cheng [21] et al. conducted an in-depth data-driven analysis of the correlation between DRAM errors and server failures based on memory CE error logs generated by mcelog. For the first time, they incorporated One-hot encoding of server memory hardware configurations into feature extraction and designed a complete machine learning-based workflow for server failure prediction. The performance of different machine learning modeling approaches with this feature set is validated through large-scale experiments, and it is found that tree-based models typically perform better in predicting CE drive failures, but the 63% F1 score still reflects the limitations of the current feature representation capability.

In addition to analyzing the relationship between log and sensor data and DRAM errors, Du et al [22] introduced a new fault prediction mechanism through an online learning approach to address the difficulty of adapting traditional static modeling methods to dynamic error patterns. Their study proposes a prediction framework based on historical error observations, which realizes the dynamic discovery of implied patterns by evaluating the similarity between current error observations and historical observations through an innovative kernel function method. Although the method proposes a new memory management strategy by integrating hardware loss level and runtime context, the 65% accuracy indicates that there is still much room for improvement in the generalization ability of the model. The operational state of a server has a significant impact on memory failures, where the load condition is a key indicator of the operational state. In this research direction, X. Wang et al [23] thoroughly investigated the mechanism of workload impact on DRAM failure. They designed an innovative HiDEC framework to accurately represent DRAM access patterns and improve the prediction performance with the help of a decision tree-based model. Their study focuses on the mechanism of the impact of load conditions on server operating states and DRAM failures, and demonstrates through detailed experiments that macro and micro features play an important role in improving prediction performance. Although their model enabled an 80% accuracy in DRAM failure prediction, it failed to delve into the complex interactions between load characteristics and hardware configuration.

In an in-depth study at the system architecture level, E. Basement [24] et al. achieved the first accurate prediction of the probability of DRAM errors in unaccessed locations by utilizing massive amounts of data from a supercomputer and employing statistical machine learning methods. Their work not only strongly supports the expected physical behavioral characteristics of DRAM hardware, but also provides a real-time error prediction mechanism, revealing that relatively simple statistical models can effectively predict future errors based on historical data. However, this statistical modeling approach is too simplified to cope with the complexity and variability of system states in real-world scenarios. Based on this research, Q. Yu et al [17] proposed the HiMFP framework for multilayer memory systems, which is mainly used to reduce the VM outage problem caused by irreparable errors (UCE). Their study not only focuses on the UCE prediction problem, but also innovatively combines the prediction results with memory recovery techniques, which provides a brand new idea to improve system stability. Experimental results show that their model achieves 83% accuracy and 68% F1 score, which is a significant improvement over previous studies, but the method is mainly designed for specific VM scenarios, and the generalizability is still limited. In the cloud computing environment, P. Zhang et al [25] thoroughly studied the cloud node unavailability problem and developed an integrated XBrainM system by introducing innovative spatio-temporal features and NURR metrics. The system cleverly integrates ML models and traditional rule-based methods for prediction, and finally achieves an F1 score of 73%, but there is still room for improvement in terms of completeness of feature expression and model generalization ability.

Although the aforementioned studies have introduced a wealth of methodological and technological innovations to the field of memory failure prediction, yet in-depth analysis reveals that these studies still have limitations in three main areas. First, at the model construction level, existing research overly relies on a single classifier approach, which not only results in a model that is susceptible to data noise, but also faces the inherent challenges of machine learning such as severe overfitting and underfitting, as well as the lack of an effective model integration mechanism, which makes it difficult to merge the strengths of different classifiers, and the interpretability of prediction results is also poor. Second, at the level of feature engineering, research is often limited to single-dimensional data analysis, failing to form a complete multi-dimensional feature system, and unable to fully explore the complementary and synergistic effects between multi-source data; especially in the expression of time-series features, the existing methods are difficult to effectively portray the long-term evolution law of the error pattern, and the research on the interaction between hardware configuration and operation state is not deep enough. Third, at the level of application scenarios, the model design generally fails to fully consider the complexity of the system state in the actual environment, and the modeling of dynamically changing characteristics is insufficient, making it difficult to adapt to the load fluctuations; at the same time, most of the methods are designed for specific scenarios, with insufficient generalization and scalability, and the optimization of the balance between the prediction performance and the system overhead still needs to be improved. In order to break through these limitations, the introduction of an integrated learning framework becomes a solution with great development potential. By systematically integrating the advantageous features of multiple classifiers, this approach is expected to significantly improve the model’s ability to capture complex data patterns while effectively overcoming the inherent limitations of a single classifier. Meanwhile, through the deep fusion of multi-source data and feature interaction analysis, it can not only enhance the robustness of the model, but also improve the interpretability of the prediction results, which opens up a brand-new development direction for the research of memory fault prediction.

## The proposed method

Given the shortcomings in the prediction performance of individual classifiers, we propose an integrated learning approach based on collaborative decision making with adaptive thresholding, aiming to form a powerful classifier to improve the prediction performance of memory faults by integrating machine learning models (including Random Forest, LightGBM, and XGBoost) that have excelled in the prediction of CE-driven faults in server memory.

**Fig 1:**
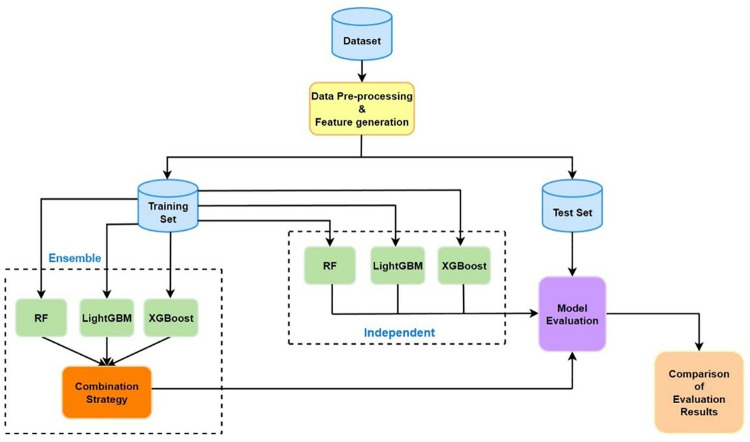
Proposed CE-driven failure prediction approach.

Fig 1 illustrates our proposed method for predicting server memory CE (Correctable Error) driven failures. First, we preprocessed the dataset to balance the ratio between positive and negative samples. Then, based on the feature extraction method for memory CE error behavior [21], we extracted features from DRAM error logs and memory configuration files. For each server, we formed a dataset containing 52 features. Next, we divided the data into training and test sets. The training set was used to train both individual classifiers and an ensemble model composed of these classifiers. The base classifiers in the ensemble model were integrated using a collaborative decision method based on adaptive thresholds. For each individual classifier, we used grid search to select the optimal classifier parameters. Similarly, for the ensemble model, we also used grid search to find the optimal parameters, namely the optimal combination parameters of each base classifier and their corresponding decision impact factors, and dynamically adjusted the decision threshold based on the principle of optimal F1 score in the model’s final output. After parameter adjustment, we compared the performance of our proposed ensemble model with that of the single best base classifier. This method aims to improve the accuracy and reliability of memory CE drive failure prediction by combining decisions from multiple classifiers.

Through ensemble learning, multiple high-performance learning models can be effectively integrated to significantly improve overall performance. In the field of server memory failure prediction, previous research has shown that tree-based prediction models outperform other machine learning models, including deep learning models, and generally exceed other models by about 20% in performance metrics. According to previous experimental results, the Random Forest model has demonstrated excellent performance across different datasets, showing strong generalization ability, and helps reduce the risk of overfitting due to its characteristic of averaging predictions from multiple trees. In the extracted high-dimensional feature data, XGBoost showed higher precision compared to Random Forest. On the other hand, LightGBM performed comparably to XGBoost in terms of precision while offering faster training speeds compared to XGBoost. The following sections 3.1 to 3.3 will detail the principles of these three algorithms.

### Random forest

**Fig 2:**
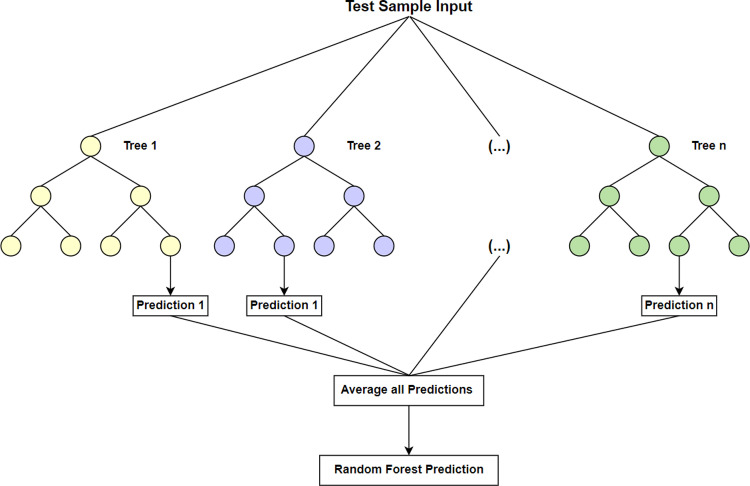
Structure of random forest model.

Random forest is an ensemble learning model based on decision trees [26]. It performs prediction by constructing and synthesizing the prediction results of multiple decision trees, which improves the precision and stability of prediction. Each decision tree is trained independently on a randomly selected subset, and this randomness not only increases the diversity of the model, but also improves the robustness and generalization ability of the model. Each decision tree consists of a root node and multiple child nodes, each of which is associated with a feature variable. During the construction of the decision tree, the splitting of nodes is performed based on the impurity minimization criterion, ensuring that each decision tree is able to make the most accurate prediction. Eventually, the predictions of all decision trees are integrated by methods such as majority voting strategy or weighted average to generate a global and integrated prediction, which improves the precision and robustness of the overall prediction.Fig 2 demonstrates the structure of the random forest model.

### XGBoost

**Fig 3:**
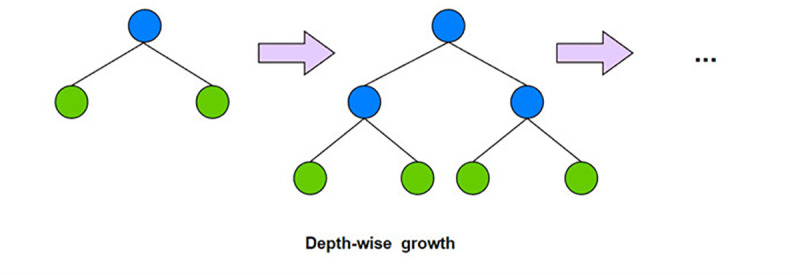
Structure of XGBoost.


The XGBoost algorithm [27] is an ensemble learning model based on gradient boosting decision trees. It works mainly by iteratively training a series of decision trees, where each new decision tree tries to correct the prediction errors of all previous trees. In XGBoost, each decision tree is a regression tree [28], and the final prediction results are obtained by weighted superposition of the predictions of multiple regression trees. As shown in Fig 3, XgBoost uses Depth-wise generation of decision trees, splitting the leaves of the same layer at the same time, so as to perform multithreaded optimization, which is not easy to overfitting. At the same time, XGBoost also supports custom loss functions and regularization terms, which can further improve the generalization ability and robustness of the model. The overall objective function of its model is:

$$L\left(\phi \right)=\sum_{i}l\left(y_{i},\hat{y_{i}}\right)+\sum_{k}\text{Ω}\left(f_{k}\right)$$ (1)

where $L\left(\phi \right)$ is the expression on linear space, i is the ith sample, k is the kth tree, $\hat{y_{i}}$ is the predicted value of the ith sample $x_{i}$, and $\sum_{k}\text{Ω}\left(f_{k}\right)$ denotes the complexity of the k-tree.

### LightGBM

**Fig 4:**
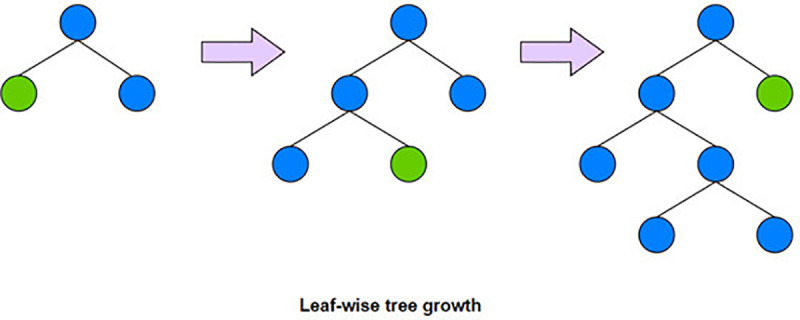
Structure of LightGBM.

LightGBM algorithm [29] is a machine learning algorithm based on gradient boosted trees, developed by Microsoft, which has a wide range of applications in machine learning and data mining.The advantages of LightGBM over traditional gradient boosted tree algorithms in terms of training and prediction speeds are mainly due to the following improvements: histogram-based decision tree algorithms reduce the computational complexity and the difficulty of feature processing; Vertical parallelization training algorithms (GOSS and EFB) improve the model training efficiency, especially for large-scale datasets; Leaf-wise growth strategy accelerates the directional search for loss function reduction; and memory optimization technique reduces the memory consumption and improves the data reading efficiency. Fig 4 shows the Leaf-wise growth strategy.

### Adaptive threshold-based system decision-making approach

The core idea of ensemble learning model as a machine learning method [30] is to obtain superior performance over a single model by integrating the outputs of multiple individual learners, including higher prediction precision, stronger generalization ability, and better robustness. Among them, the voting method, as one of the ensemble learning models, has been widely adopted and applied to various tasks. Voting methods can be mainly categorized into two main types: hard voting and soft voting. In the hard voting strategy, each base classifier independently labels the samples with categories, and the category of the one with the most votes is finally used as the output of the integrated model. However, this type of voting ignores the differences in the classifiers’ confidence levels for different categories. In contrast, the soft voting strategy allows the base classifiers to output probability estimates for each category. The final decision of the ensemble model is based on a weighted average of the probability outputs of all classifiers. This strategy takes full advantage of the confidence information provided by the classifiers and therefore typically performs better when dealing with data with noise and uncertainty.

The collaborative decision-making method is an advanced strategy within integrated learning that aims to enhance overall prediction performance by effectively leveraging the strengths of multiple models. Unlike conventional ensemble methods, which typically aggregate predictions, this approach assigns varying decision influence factors to each model, allowing their contributions to be weighted according to their performance and relevance in the given context. This dynamic weighting mechanism recognizes that different models excel in different aspects of the prediction task, enabling a more nuanced integration that optimally harnesses the capabilities of each model.In this framework, each base model is treated not merely as a predictor but as a decision-making agent, with its influence on the final output determined by its reliability and accuracy. The decision influence factor assigned to each model reflects its performance in specific conditions, such as precision, recall, or F1 score, ensuring that models that consistently perform well have a greater impact on the final decision. Conversely, models that demonstrate weaker performance or greater variance in their predictions are allocated lesser influence, minimizing their potential to introduce noise or bias into the decision-making process.The optimization of these decision influence factors is central to improving prediction accuracy and stability. The process involves a careful evaluation of each model’s performance under varying conditions and identifying which models excel in particular areas. By adjusting the influence factors accordingly, the method ensures that the final prediction represents a weighted synthesis of the models’ strengths, rather than simply an average of their outputs. This strategy not only enhances the overall prediction accuracy but also improves the stability of the model across different data subsets and input scenarios.Moreover, the adaptability of this method makes it particularly well-suited for complex and high-dimensional datasets, where different models may specialize in recognizing distinct patterns or feature relationships. For instance, a model with strong performance in time-series forecasting may be given greater influence when dealing with historical data, while a model that excels in processing high-dimensional features might dominate in tasks involving hardware configuration data.In sum, the collaborative decision-making method provides a sophisticated and flexible framework for integrating diverse models, ensuring that the final prediction is a balanced and weighted combination of all models’ outputs. By optimizing decision influence factors, this approach effectively mitigates the risk of overfitting and reduces model bias, resulting in a more robust and generalized prediction process. This method is particularly beneficial in complex prediction tasks, such as memory fault prediction in data centers, where the ability to adapt to various data patterns and models is essential.

**Fig 5:**
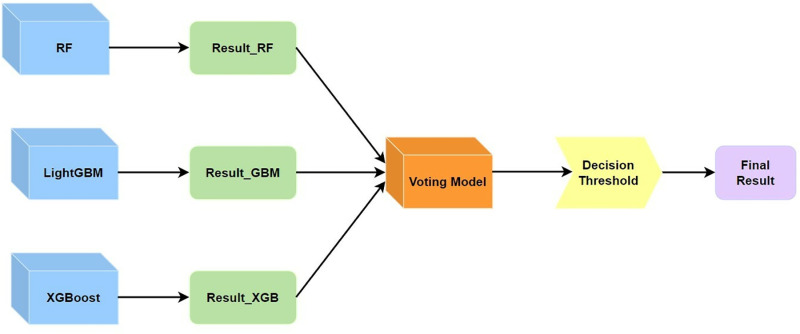
Weighted voting model.

In the field of memory failure prediction, Random Forest, with its excellent generalization ability and reduced risk of overfitting, is able to strike a good balance between precision and recall, and thus is prone to achieve good F1 scores. While XGBoost shows high precision in high-dimensional feature data. Compared with XGBoost, it possesses a considerable precision rate and at the same time, the training speed is faster. We collaborate the above three models for decision making in order to utilize their respective strengths in integrated learning and improve the performance of the prediction model by means of dynamically adjusting the decision thresholds of the model outputs. The proposed model is shown in Fig 5.

In this strategy, each base model predicts the sample and outputs probabilities for each category. These probabilities are then weighted and averaged to obtain an average probability for each category. The output of weighted voting is shown in equation (2):


$$H\left(x\right)=\underset{j}{cargmax}\sum_{i=1}^{T}\omega_{i}h_{i}^{j}\left(x\right)$$ (2)

where $\omega_{i}$ is the weight of the individual learner $h_{i}$ which is usually required to be $\omega_{i}⩾0,\sum_{i=1}^{T}\omega_{i}=1$.

The output of the model usually needs to be compared with a predetermined threshold when making decisions. When the weighted average probability of a category exceeds this threshold threshold, the system will select that category as the final prediction result. In this process, the selection of the judgment threshold has a significant impact on the prediction result of the classification model. In a binary classification problem, the output probability values of the model are mapped and used by the decision process to determine whether the sample belongs to the positive or negative category. The setting of the judgment threshold plays a key role here in determining which probability values are categorized as positive categories and which are categorized as negative categories. The choice of judgment threshold directly affects the sensitivity and specificity of the model and when the final category output is performed, the output formula is shown in equation (3):


$$y\left(x\right)= \{\begin{array}{c} 1ifH\left(x\right)\geq Threshold\\ 0ifH\left(x\right)<Threshold \end{array}$$ (3)

where Threshold is the best judgment threshold of the classifier for that category.

In the adjudication phase, the adaptive thresholding mechanism processes the integrated model outputs to find the optimal adjudication threshold, which is selected based on a user-specified performance metric. For each integrated model output, the adaptive thresholding mechanism traverses different judgment thresholds within a specified range (typically from 0 to 1). For each threshold, the system calculates the performance of the model at that threshold based on a user-selected performance metric (e.g., F1 score). The system identifies at which threshold the model’s performance metrics are optimal. For example, under the optimal F1 score policy, the system will find the threshold that maximizes the F1 score. After finding the optimal threshold, the system will apply the threshold to the corresponding integrated model as the final judgment threshold.

This adaptive mechanism can adapt more flexibly to the characteristics of different tasks and datasets, improving the model’s prediction performance so that it can achieve the best performance in various contexts. Compared to the fixed-threshold approach, this adaptive thresholding mechanism can better adapt to the differentiation of model outputs, thus enhancing overall prediction performance.

One of the main advantages of the adaptive thresholding mechanism is its ability to balance precision and recall more effectively across different datasets. In datasets with imbalanced classes, for instance, a fixed threshold may result in suboptimal performance, especially if it does not align with the distribution of the classes. By dynamically adjusting the threshold for each integrated model output based on the selected performance metric (such as F1 score), the adaptive thresholding mechanism can optimize both precision and recall more accurately.For example, in a highly imbalanced dataset where false positives are more costly than false negatives, the adaptive thresholding mechanism can adjust the threshold to maximize precision and minimize false positives. Conversely, in cases where recall is more critical (e.g., identifying potential failures), the threshold can be adjusted to ensure that more positive instances are captured, even at the cost of some precisionThis flexibility allows the system to adapt to different datasets’ characteristics, balancing the trade-off between precision and recall according to the specific needs of the application. As a result, the adaptive thresholding mechanism improves the model’s robustness and predictive accuracy, especially when dealing with datasets that exhibit significant class imbalance or differing distributions of positive and negative instances.

## Results and discussion

The dataset used in our work is a large-scale dataset released by Aliyun Tianchi Labs for predicting server failures due to DRAM errors.The dataset was collected from more than 250K servers and 30,000 DIMMs over an eight-month span at Alibaba.The dataset consists of three main data type types, which include mcelogs logging more than 700,000 DRAM errors, thousands of trouble tickets describing server failures due to DRAM errors, and hardware configuration inventory logs.

Mcelog. mcelog is a tool for checking DRAM hardware errors on Linux systems. The tool is mainly used for analyzing and logging CE errors. When the hardware detects an error, the system generates MCE events, which can be read by mcelog to provide corresponding reports, including Server ID, DIMM ID, Rank ID, Bank ID, Row ID, Column ID, and the time the error occurred. ID, Column ID, and the time the error occurred. In total, our error logs recorded 75.1 M CE from 30,496 servers (both normal and faulty servers) over an 8-month period.

**Fig 6:**
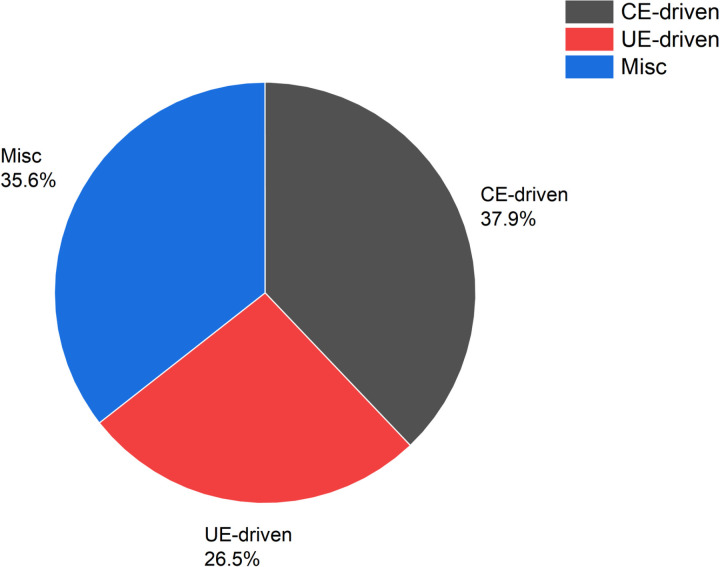
The portion of different kinds of failures.

Trouble Tickets. Trouble Tickets record the server failure records issued by the centralized maintenance system, which include the server ID, failure category, and the timestamp of the failure. A total of 3017 trouble tickets were collected over a period of 8 months.Since our fault prediction is CE-driven fault prediction through CE features, among all the trouble tickets we only cared about the ones that had at least one CE error prior to the server failure, which amounted to 2137 trouble tickets in total. Among these fault orders there are 809 CE-driven faults,567 UE-driven faults and 761 other faults. Overall, the dataset we adopt contains diverse samples covering server memory fault data of different models, brands and usage environments, ensuring the richness and diversity of the samples with good generalizability. Fig 6 shows the percentage of different types of faults.

Inventory List. The Hardware Configuration List records the Server ID, Server Manufacturer, DRAM model and DIMM count. Our dataset contains 7 different DRAM models from 4 server manufacturers and 4 different DIMM count configurations, 8, 12, 16, 24, respectively. The hardware configuration list helps us to analyze the relationship between DRAM failures and server hardware configurations.

### Feature extraction

Since the number of positive samples in the experimental dataset is much less than the number of negative samples (we labeled the server samples that generated CE driver failures as positive samples and the rest as negative samples), we labeled the additional samples from the day before each server failure as positive samples during the feature extraction process. To address the class imbalance, we used downsampling to balance the ratio of positive to negative samples to achieve a ratio of 1:50. Previous research [17] has shown that this downsampling ratio achieves the best prediction accuracy, and this research has also shown that adding positive samples one day before each failure does not significantly affect the model performance, and that this approach does not compromise the predictive power of the model.

After the data preprocessing is completed, we extract features for each server within the feature window.Our feature extraction utilizes a feature extraction method based on DRAM error logs and hardware configuration [21]. We define the feature window as the time interval before prediction, and since CEs tend to occur within a short period of time, we set the feature window to 5 minutes to include the most recent CEs. The 5-minute window is chosen because it effectively captures the accumulation of correctable errors (CEs) that occur within a short time frame, which is typical for CE-driven failures. A smaller window (e.g., 1 minute) might fail to capture the full trend of CE occurrences, potentially missing critical fault patterns, while a larger window (e.g., 10 minutes) could introduce delays, compromising the timeliness of fault detection. By keeping the feature and prediction windows consistent at 5 minutes, we ensure that the model both captures relevant historical data and responds quickly to real-time changes, thus enhancing prediction accuracy and efficiency. Within the feature window, we extracted a total of 52 features from 4 feature groups per server.

**Fig 7:**
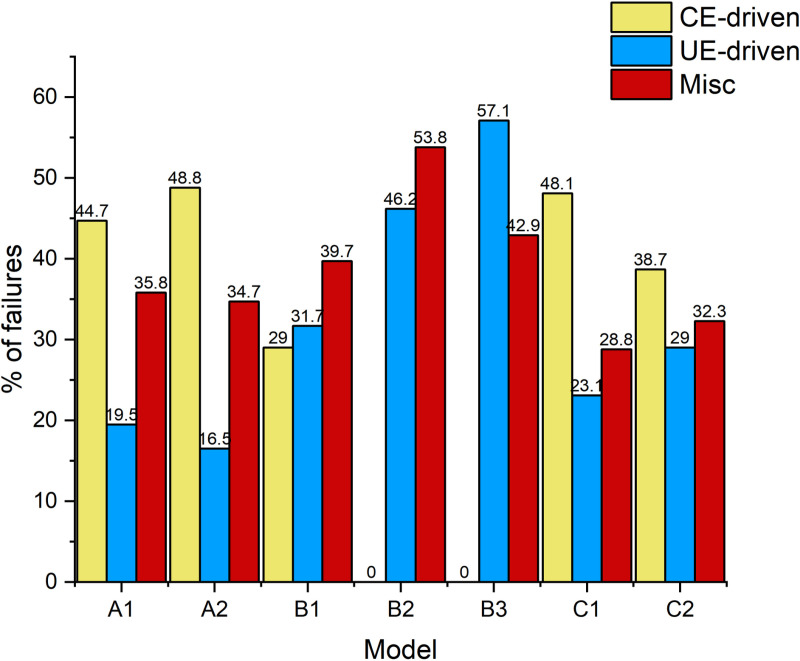
DRAM failures of different models.

**Fig 8:**
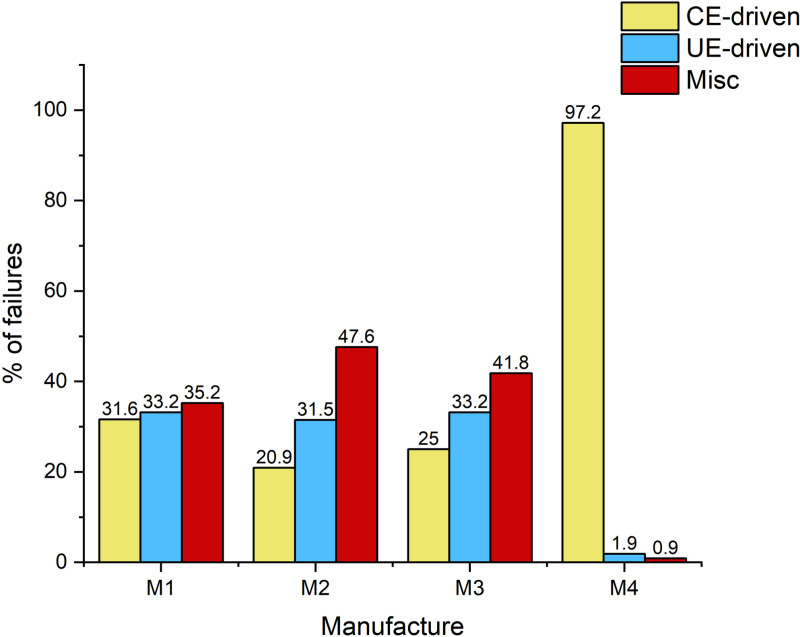
DRAM failures of different server manufactures.

**Fig 9:**
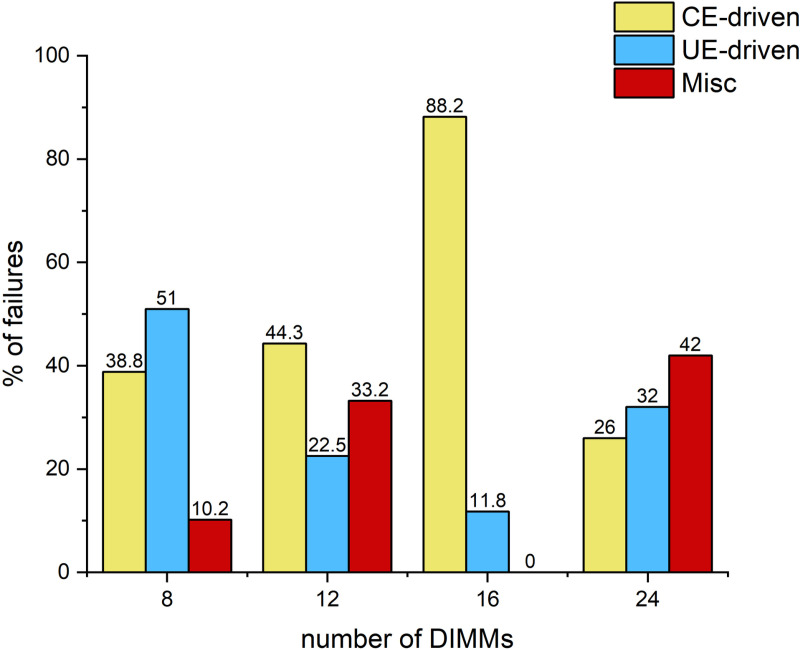
DRAM failures of different server manufactures.

Configuration features, Configuration features play a key role in server memory failure prediction, and previous studies have clearly demonstrated that using the configuration of server memory as a feature, especially in random forest models, significantly improves the model’s prediction precision. For example, the F1-Score, Precision, and Recall of the random forest model improved by 23.5%, 18.7%, and 29.7%, respectively, when the model was trained using data containing configuration features. Therefore, we analyzed the memory failures in detail for different memory models, server manufacturers, and the number of DIMMs, and Fig 7 shows the server memory failures for different configurations. According to the results shown in Fig 7, the highest percentage of CE-driven failures occurred in servers of model A2, while servers of models B2 and B3 did not experience CE-driven failures; Fig 8 demonstrates memory failures experienced by servers produced by the service manufacturer M4, the vast majority of which were CE-driven failures, while servers from other manufacturers experienced CE-driven failures to a lesser extent; and Fig 9 presents memory failures with the highest percentage of servers experiencing CE-driven failures when the number of DIMMs is 16.

Combining the previous studies and the above data analysis, we learned that the configuration of server memory, including DRAM model, number of DIMMs, and manufacturer, has a considerable impact on the frequency of servers experiencing CE-driven failures. In order to fully utilize this critical configuration information, we use One-hot coding to convert configuration features such as DRAM model, DIMM quantity, and server manufacturer into numerical form to form corresponding configuration features. This step helps to incorporate the qualitative configuration information into the model training and further improves the accurate memory failure prediction of servers.Additionally, the diversity of our dataset, which includes different hardware configurations, plays a crucial role in enhancing the model’s ability to generalize across diverse environments. By including a range of DRAM models, DIMM counts, and manufacturers, we ensure that the model is trained on data reflecting various server configurations. This allows the model to capture fault patterns related to different hardware setups and adapt to various data centers. The inclusion of these configuration features helps the model generalize well, making it more adaptable to different environments and increasing its reliability when applied to new, unseen data from other data centers.

Counter Features, we extract features reflecting the numerical amount of CE errors within the feature window, such as the total count of CEs, hard errors, soft errors, read errors, cleanup errors, and the average time between errors (MTBE). The CEs are directly counted from the mcelog within the feature window. Hard and soft errors are categorized based on their occurrence frequency: when multiple CE errors occur in the same unit during the cleanup cycle (which is 24 hours in this study), they are classified as hard errors; otherwise, they are soft errors. Read errors and cleanup errors are categorized by the source of the errors reported by mcelog. Cleanup errors are detected by the memory cleaner, and read/write errors are caused by DRAM read/write operations. The MTBE feature represents the average time between a pair of neighboring CEs, providing valuable temporal information.

For component-specific characterization, we count the number of CEs associated with each of the seven types of component failures: socket, channel, bank, row, column, and random errors. Additionally, we classify multiple errors occurring within the same unit. For instance, more than one error within the same cell is classified as a Cell failure, more than one Cell failure in the same column is considered a Column failure, and so on. These hierarchical failure patterns are crucial for understanding the memory subsystem’s failure modes. Furthermore, we count the number of components (excluding random errors) affected by each failure.

Statistical Characterization, we calculate the mean, median, and standard deviation of the number of CEs for each of the six component faults (socket, channel, bank, row, column) in addition to random errors. These statistical features help quantify the variability and distribution of CEs, which can be valuable for distinguishing between normal and abnormal memory behavior.

By selecting features that capture both memory CE error behavior and memory configurations, we enhance the model’s ability to understand and represent the data effectively. The feature set incorporates temporal information, such as the five-minute window for error occurrence and the MTBE, which are important for identifying patterns over time. The relationships between these features, including those related to the memory components and error types, provide a holistic view of memory behavior. This interconnectedness helps the model learn and detect complex patterns, ultimately improving the model’s performance, efficiency, and robustness.In conclusion, the features we’ve selected are crucial for representing the system’s behavior, including both the temporal dynamics and the underlying structural patterns. We believe that this comprehensive feature set enables the model to effectively capture memory failure characteristics, which enhances its predictive capability.

### Evaluation metrics

The results obtained from the experiments are analyzed through the Precision, Recall, and F1-Score metrics.TP corresponds to the true case, which represents the number of actual faulty servers that are predicted to be faulty, TN corresponds to the true-negative case, which represents the number of actual healthy servers that are predicted to be faulty, FP corresponds to the false positive case, which represents the number of actual healthy servers that are predicted to be faulty, and FN corresponds to the false-negative case, which represents the number of actual healthy servers that are predicted to be faulty. servers, and FN is a false negative example indicating the number of servers that are actually faliure prediction servers but are predicted to be healthy servers.

Precision denotes the precision of the model in CE-driven memory failure prediction, i.e., the percentage of servers that actually have CE-driven failures that are correctly predicted by the model, which is calculated as shown in equation (4).


$$Precision=\frac{TP}{TP+FP}$$ (4)

Recall indicates the completeness of the model’s CE-driven memory failure prediction, i.e., the proportion of CE-driven faulty servers correctly predicted by the model to all actual CE-driven faulty servers, which is calculated as shown in equation (5).


$$Recall=\frac{TP}{TP+FN}$$ (5)

The F1-score represents the model’s comprehensive performance between precision and recall. It is the reconciled average of precision and recall, which balances the precision and completeness of the model, and is calculated as shown in equation (6).


$$F1-score=\frac{2*Precision*Recall}{Precision+Recall}$$ (6)

In practice, high Precision means that the model can accurately distinguish between faults and normal states, especially in memory fault prediction, which can effectively avoid misjudging normal samples as faults. This not only reduces the inspection and maintenance caused by false positives, but also reduces the waste of resources and improves the efficiency of operation and maintenance. The memory fault diagnosis and data transfer mechanisms in modern data centers are quite complete, and the underlying maintenance system is usually able to safely transfer data by automated means to avoid serious impact. However, false positives (misdiagnosing a normal state as a fault) can still lead to unnecessary maintenance operations and increase O&M costs. Therefore, reducing false positives and pursuing a higher accuracy rate becomes a key goal of our optimization model. Low false positives are especially important for data centers because frequent false positives increase O&M costs and put additional pressure on the system.The F1 score, as a comprehensive evaluation metric, balances precision and recall and provides a more comprehensive performance feedback in case of data imbalance. Therefore, we pay special attention to the F1 score to ensure that the model excels in accuracy and completeness. During the model design process, we will focus on high precision and low false positives to improve prediction performance by optimizing the balance of false positives and false negatives. This not only reduces O&M costs, but also improves system stability and reliability, enabling the model to demonstrate greater adaptability and utility in complex, dynamic environments.

### Experiment and result

The experimental platform on which our work is based is a computer equipped with Intel Core i7-12700H CPU@2.7GHz and 16 GB of RAM, the operating system is Windows 11, and all models are implemented in a Python environment.

In order to evaluate the effectiveness of server faliure prediction, we divide the 8-month experimental data into a training set and a test set, where the data from January to June is the training set for training the prediction model, and the data from July to August is used as the test set for testing and evaluating the model’s performance metrics. In order to verify the performance improvement of the fusion models, RandomForest, LightGBM and XGBoost models are constructed for server memory CE-driven failure prediction, and each model is tuned to achieve its optimal performance through grid search. The main parameters of each model are finally obtained as shown in Table 1 below:

**Table 1:** Best parameters of base classifiers.

Model	n _ est	max_d	min_samples_split	learning_rate	colsample
RF	60	35	12	\	\
LightGBM	40	10	10	0.01	\
XGBoost	400	15	\	0.1	0.7

After tuning each individual model to maximize its performance and obtaining the relevant results, we integrated the individual models using an adaptive thresholding-based collaborative decision-making approach to obtain strong classifiers. Initially, we integrated two of the three classifiers—Random Forest, LightGBM, and XGBoost—and then proceeded to integrate all three models to verify the effect of different collaborative decision-making strategies on model performance.In our experiments, we observed that different weight configurations for the base classifiers had a significant impact on the performance of the integrated model. For instance, when we set the weights of Random Forest, LightGBM, and XGBoost to 0.4, 0.4, and 0.2, respectively, the model achieved an F1 score of 0.74, Precision of 0.80, and Recall of 0.68. However, adjusting the weights to 0.3, 0.6, and 0.1 resulted in an F1 score of 0.72, Precision of 0.74, and Recall of 0.70. This demonstrated the sensitivity of model performance to the classifier weights.Based on this observation, we used a grid search method to fine-tune the decision impact factors of each classifier, treating these factors as hyperparameters for the integrated model. We varied the decision impact factors in steps of 0.01, ensuring that the sum of the weights for all three classifiers always equaled 1. This optimization process allowed us to refine the model and improve its performance.Additionally, the adaptive thresholding mechanism was used in conjunction with grid search to find the best threshold for each classifier, aimed at maximizing the F1 score while balancing Precision and Recall. By applying the adaptive thresholding mechanism with a step size of 0.01, we could dynamically adjust the decision thresholds to optimize the trade-off between Precision and Recall for each individual model. This further enhanced the overall performance of the integrated model.After training to find the best combination of decision impact factors, we found that integrating all three models (Random Forest, LightGBM, and XGBoost) resulted in better performance than integrating just two models. The final decision impact factors were 0.55 for Random Forest, 0.22 for LightGBM, and 0.23 for XGBoost. The parameters of the integrated model are summarized in Table 2 below:

**Table 2:** Parameters of base classifiers in ensemble model.

Model	n _ est	max_d	min_samples_ split	learning_rate	colsample	weight
RF	50	25	10	\	\	0.55
LightGBM	35	10	10	0.01	\	0.22
XGBoost	300	11	\	0.1	0.7	0.23

To validate the effectiveness of the proposed integration framework, we conduct detailed ablation experiments. Table 3 shows the experimental results for different model combinations. First, we evaluate the individual performance of the three base classifiers (Random Forest, LightGBM, and XGBoost). The results show that Random Forest performs best in terms of F1 score (70.5%), while LightGBM leads in terms of accuracy (77.7%).

**Table 3:** Ablation study: performance comparison of different model combinations.

RandomForest	LightGBM	XGBoost	F1 Score(%)	Precision(%)	Reacll(%)
√	×	×	70.5	71.4	**69.6**
×	√	×	64.5	77.7	55.1
×	×	√	69.8	73.3	66.6
√	√	×	70.1	83.3	60.6
√	×	√	73.8	80.1	68.4
×	√	√	70.7	81.7	62.4
√	√	√	**74.1**	**84.5**	66.6

Next, we validated the effect of dual-model integration. The experimental results show that the integration of Random Forest with XGBoost is able to improve the F1 score to 73.8%, while the integration of Random Forest with LightGBM achieves 83.3% in terms of accuracy rate, which is an improvement of 5.6 percentage points compared to a single model. This demonstrates a good complementarity between the different base classifiers.

Finally, we validate the performance of the complete three-model integration scheme. The results show that the three-model integration achieves the optimal performance in both F1 score and accuracy, reaching 74.1% and 84.5%, respectively. Compared with the optimal single-model scheme, the F1 score and precision rate are improved by 3.6 and 6.8 percentage points, respectively; compared with the optimal two-model integration scheme, these two metrics are still improved by 0.3 and 1.2 percentage points. In terms of recall, the three-model integration reduces 3.0 and 1.8 percentage points compared to the single- and two-model optimal results, respectively.

Table 4 presents the results of comparing our integrated model with other state-of-the-art models in the domain. From the table, it can be observed that our integrated model outperforms the other models in both F1 score and accuracy. Specifically, the HIFMP model [17] has a similar accuracy to our model, but its F1 score is lower by 10 percentage points, primarily due to a significantly lower Recall. The model based on XBrainM-Rule+XGB [25] achieves a high Recall of 78%, but its accuracy is substantially lower than that of our collaborative decision-making model, resulting in an F1 score that is 2 percentage points lower.

**Table 4:** Comparison with state-of-the-art models.

Model	F1 Score(%)	Precision(%)	Reacll(%)
RandomForest[21]	62.9	59.6	66.8
HIMFP[24]	63	83	57
XBrainM-Rule^+^XGB[25]	72	67	**78**
LSTM	64.3	61.2	67.4
The proposed model	**74.1**	**84.5**	66.6

We also implemented our own LSTM model as part of the comparison. The LSTM model’s performance in our memory failure prediction task was slightly worse than our integrated model, with F1 score, Precision, and Recall of 0.6428, 0.6117, and 0.6774, respectively. We believe this lower performance is due to two factors: first, the short-term, abrupt fault patterns of CE-driven failures, which LSTM struggles to capture as it is better suited for learning long-term dependencies; second, the class imbalance in our dataset, where negative samples vastly outnumber positive ones, which limited LSTM’s ability to effectively learn from the smaller number of positive samples.

Additionally, compared to the Random Forest model used in the literature [21], our model demonstrates significantly higher accuracy, with a Recall similar to that of Random Forest, and an F1 score that is 11.2 percentage points higher. These results highlight the superior performance of our integrated approach in tackling memory failure prediction tasks.

## Discussion

**Fig 10:**
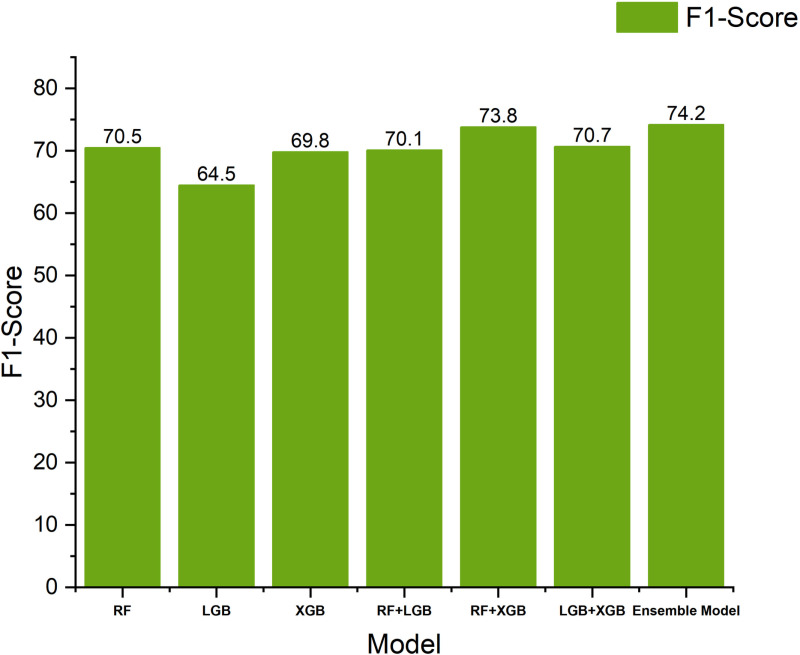
Comparison histogram of experimental F1 results of models.

**Fig 11:**
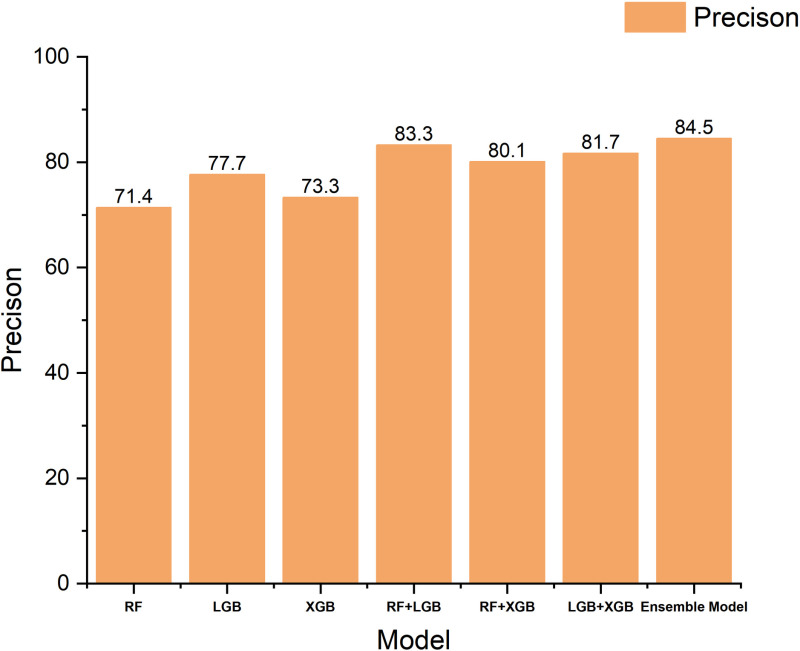
Comparison histogram of experimental precision results of models.

**Fig 12:**
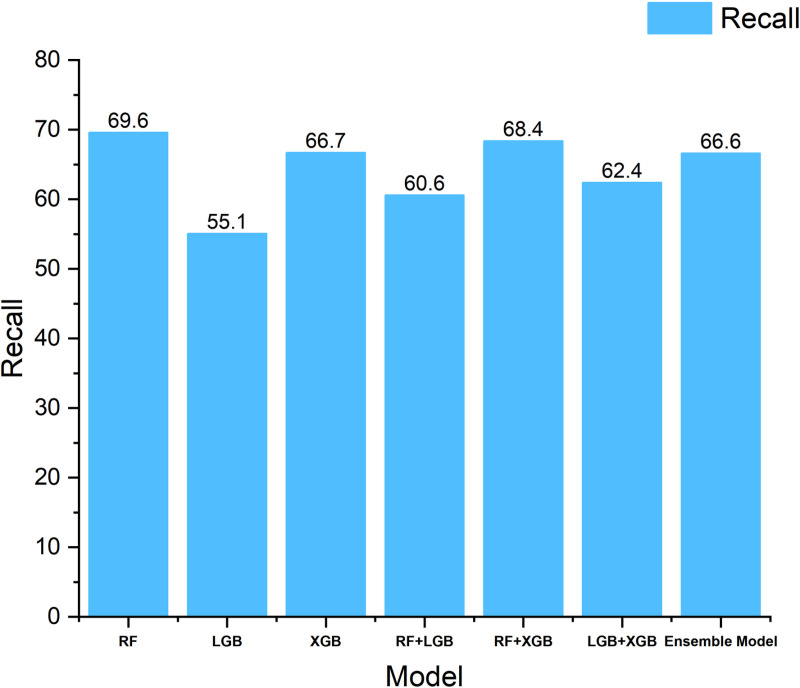
Comparison histogram of experimental recall results of models.

Figs 10–12 show the performance of different models in terms of F1 score, Precision, and Recall metrics, respectively. Through the analysis, we find that the Precision of the RandomForest algorithm is the lowest among the three base classifiers, but its F1 score is the highest. This indicates that when predicting memory CE-driven problems, the RandomForest algorithm is able to comprehensively consider the prediction precision of both positive and negative samples, thus demonstrating good overall performance. In terms of Precision, LightGBM and XGBoost perform better than RandomForest, but their F1-score are relatively low because Recall has decreased to a certain extent compared to RandomForest, reflecting the fact that Boosting-based algorithms have some deficiencies in memory CE-driven failure prediction integrity. reflecting the shortcomings of the Boosting-based algorithm in predicting the integrity of memory CE-driven faults. When the two base classifiers make collaborative decisions, we find that the accuracy of the integrated model is improved to some extent, and the combination of Random Forest and LightGBM achieves the best prediction accuracy, while the combination of Random Forest and XGBoost achieves the best F1 scores, and this result suggests that the Random Forest algorithm has an important role to play in improving the performance of the integrated model.

Therefore, in the adaptive thresholding collaborative decision making of the model, we assign a larger decision influence factor to RandomForest to ensure that the overall performance of the model is maintained at a high level. Meanwhile, LightGBM and XGBoost are also assigned a certain decision influence factor, which is done to improve the accuracy of the model. This decision impact factor tuning strategy is consistent with the optimal combination of decision impact factors we found during model tuning. However, compared to the Random Forest algorithm, the model recall decreases for both the integration of the two models and the integration of the three models, which indicates that the inclusion of the Boosting-based algorithm in the fusion model will affect the model prediction of the memory CE-driven failure prediction integrity. The Weigted Voting classifier obtained from the fusion of the three models has a slight decrease in F1 score, but the significant increase in precision means that the model is able to more accurately predict servers that are about to experience memory CE-driven failures, which significantly reduces false positives, helps to prevent unnecessary maintenance work and waste of resources, and helps administrators to more accurately track down and resolve problems.

**Fig 13:**
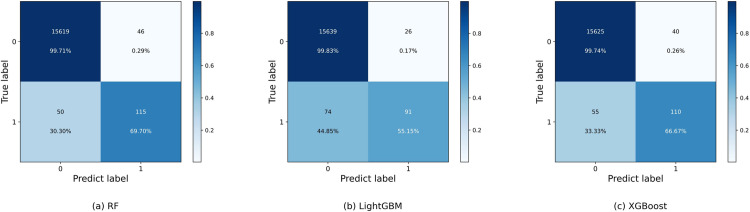
The confusion matrix of different independt classifiers.

**Fig 14:**
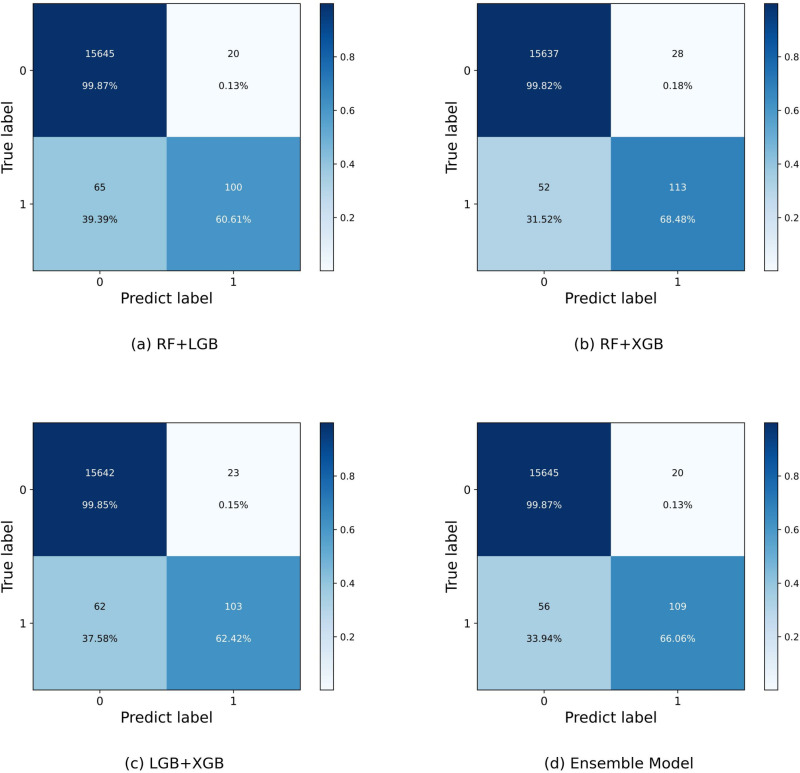
The confusion matrix of different voting classifiers.

Fig 13 and Fig 14 show the confusion matrix for different classifiers. As shown in Fig 13(a), observing the classification of RF in Fig 13(a), although the RF model successfully predicts 115 servers with impending memory CE driver failures, which is the highest among all the classifiers, at the same time, it also incorrectly predicts 46 healthy servers as faulty servers, which is the most among the three base classifiers, and thus its Precision performance is average. On the contrary, the classification of LightGBM in Fig 13(b) has an FN of 74, which is the highest among all models, indicating that LightGBM is most likely to misclassify healthy servers as faulty servers.LightGBM is a gradient boosting tree model, which progressively corrects the errors of the previous tree by constructing multiple trees. It is excellent at capturing complex patterns in data, but may be more conservative in dealing with data imbalances, especially when there are fewer minority classes (faulty servers). Specifically, LightGBM prefers to predict the majority class (healthy servers) as normal, thus ignoring the faulty servers, resulting in a higher false-negative rate. In this task, the number of healthy servers is much larger than the number of faulty servers, and this data imbalance makes it easier for LightGBM to misclassify faulty servers as normal, which affects the accurate prediction of faults. Fig 13(c) shows that XGBoost correctly predicts the number of servers about to fail in a similar way to the integrated model, but incorrectly predicts about 10 more healthy servers as about to fail than the integrated model. servers incorrectly predicted as servers that are going to fail, and thus the Precision of the XGBoost model is also lower than the integrated model.

Observe that in Fig 14(a)(b)(c), for the integrated model with two base classifiers making collaborative decisions, its correct prediction of faulty servers is similar to the three-model integration comparison, but compared to the 20 instances of FPs of the integrated model with three base classifiers, the cases in which they incorrectly predicted healthy servers as faulty servers are all greater than or equal to this value, and thus the accuracy is slightly lower. For the number of FNs i.e., cases where the model incorrectly predicts servers with memory CE-driven failures as healthy servers, RandomForest has only 50 cases while the other models are higher than this level, which results in RandomForest having the highest Recall value when RandomForest TP is also higher than the other three models.

Observing the integrated model in Fig 14(d), the combination of FP and Random Forest with LightGBM is 20, which is the lowest among all the prediction results, i.e., the integrated model incorrectly predicts samples that are actually in the negative category to be in the positive category the least, which indicates that the integrated model is less likely to misreport healthy servers as faulty servers and also successfully predicts 109 servers that are about to experience memory CE drive failures, which explains the highest Precision of the stated integrated model. Although the integrated model has the highest Precision, its Recall decreases compared to Random Forest. This change is mainly influenced by the higher false negative rate of LightGBM, which tends to misclassify healthy servers as normal, resulting in a decrease in the predictive power of the integrated model for faulty servers. Since the integrated model relies on the weighted decisions of multiple base classifiers, LightGBM’s false-negative errors affect the recall rate to some extent, resulting in a decrease in its Recall. This suggests that the integrated model misses some of the faulty servers and affects the performance of Recall while reducing false positives (FPs) and improving Precision.

**Fig 15:**
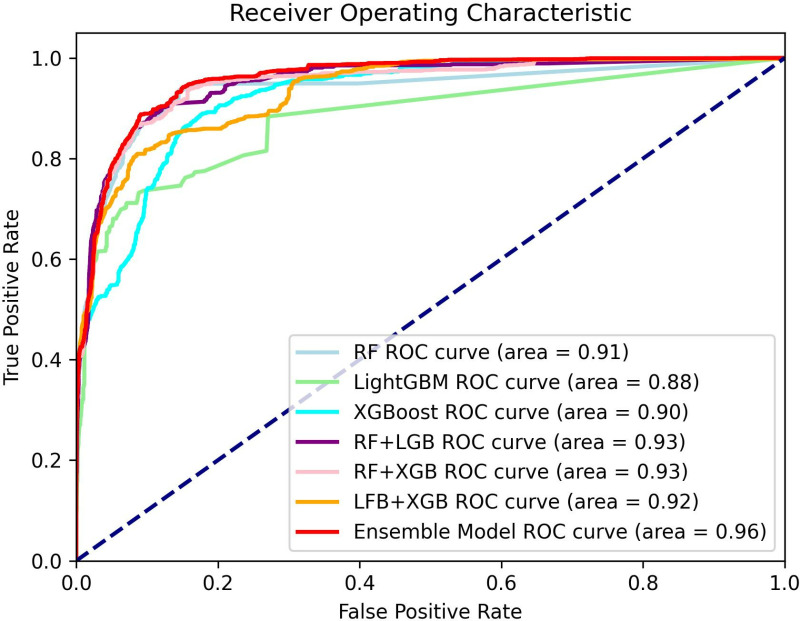
Region of operating characteristics.

Fig 15 shows the ROC curves comparing the false positivity and true case ratios of the proposed method with the base classifiers. The proposed integrated model, indicated by the red line, has better ROC performance. The integrated model by three base classifiers based on adaptive thresholding for collaborative decision making has a curve enclosure area of 0.64, which is larger than the model integrated by Random Forest, XGBoost, LightGBM, and two base classifiers. And the described integrated model achieves a higher true case rate at a lower false positive case rate, i.e., the model is able to more accurately predict servers with impending memory CE driver failures with fewer false positive cases misreporting healthy servers as faulty servers, which is in line with our expectation.

Comparing Table 1 and Table 2, we can find that when obtaining the best ensemble model, for each base classifier, its parameters are not optimal for independent prediction. This is because each model does not need to be optimal when model integration is carried out, but only needs to maximize the advantages of each model, which is needed to maximize the precision as much as possible in the case of LightGBM and XGBoost, and the Random Forest needs to provide its good performance in predicting the integrity of CE-driven memory failure prediction, complementing each other’s strengths when integrating to get the best prediction model.

**Fig 16:**
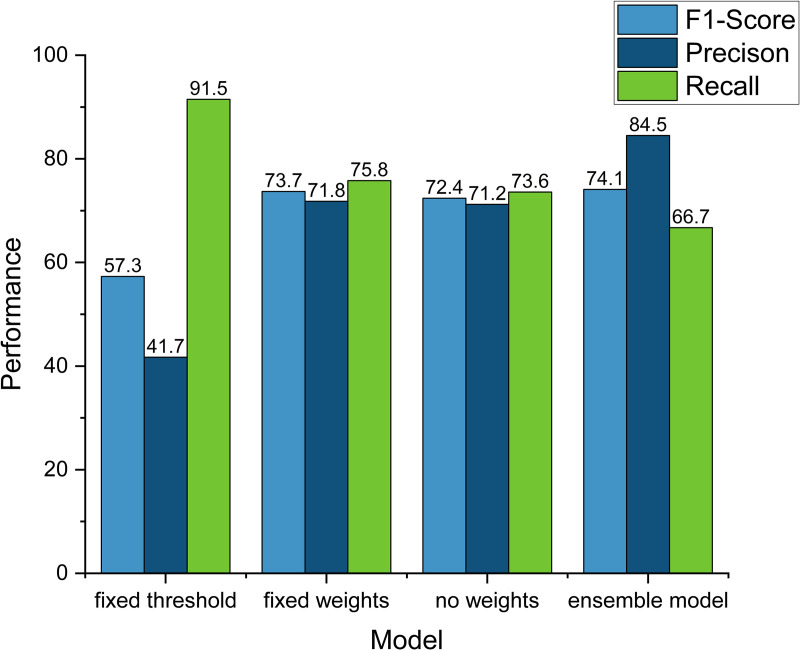
Ablation study on ensemble model configurations.

We evaluated the impact of different configurations on the performance of the integrated model through ablation experiments, the results of which are shown in Fig 16. The experiments consisted of four configurations: 1) fixed thresholding mechanism (i.e., no dynamic thresholding mechanism); 2) fixed decision factor assignment; 3) no decision factor assignment; and 4) the final integrated model (combining dynamic thresholding mechanism and decision factor optimization). The experimental results show that under the fixed thresholding mechanism, although the model’s recall reaches 91.5, the precision and F1 score are only 41.7 and 57.3, respectively, which suggests that the fixed thresholding is unable to flexibly adapt to the changes in the model’s output, resulting in insufficient overall performance. After adopting the fixed decision factor assignment configuration, the F1 score of the model is improved to 73.7, and the precision and recall are 71.8 and 75.8, respectively, which indicates that a reasonable decision factor assignment can effectively improve the model performance, but there is still a gap with the performance of the final integrated model. Without decision factor assignment, the F1 score of the model is 72.4, the precision is 71.2, and the recall is 73.6, and the results are close to those of the fixed decision factor assignment configuration, indicating that the optimization of the decision factors has a certain promotion effect on the stability of the model. Finally, the integrated model performs best in terms of F1 score, precision and recall, with an F1 of 74.1, a precision of 84.5 and a recall of 66.7, which verifies that the combination of the dynamic thresholding mechanism and the weight optimization significantly improves the overall performance while balancing the precision and recall.

In conclusion, the experimental results show that the dynamic thresholding mechanism and the optimization of the base classifier weights are the key factors to improve the performance of the integrated model. With the introduction of the dynamic thresholding mechanism, the model is able to flexibly adjust the decision threshold according to the output of the base classifiers, which overcomes the limitations brought by the fixed thresholding mechanism and enhances the adaptability and flexibility of the model. At the same time, optimizing the decision factors of the base classifiers can give full play to the advantages of each base classifier, further improving the overall performance of the integrated model. By combining these two mechanisms, our integrated model not only achieves a better balance between precision and recall, but also significantly improves the F1 score, reduces false positives, and improves the prediction accuracy. This demonstrates that the proposed integrated model is highly complete and tractable, can exhibit excellent performance on different tasks and datasets, and has strong potential for application.

**Fig 17:**
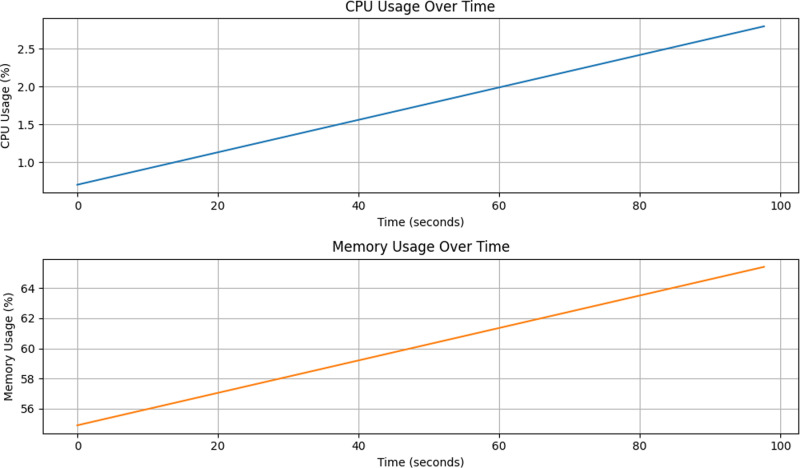
Fixed threshold model performance overhead.

**Fig 18:**
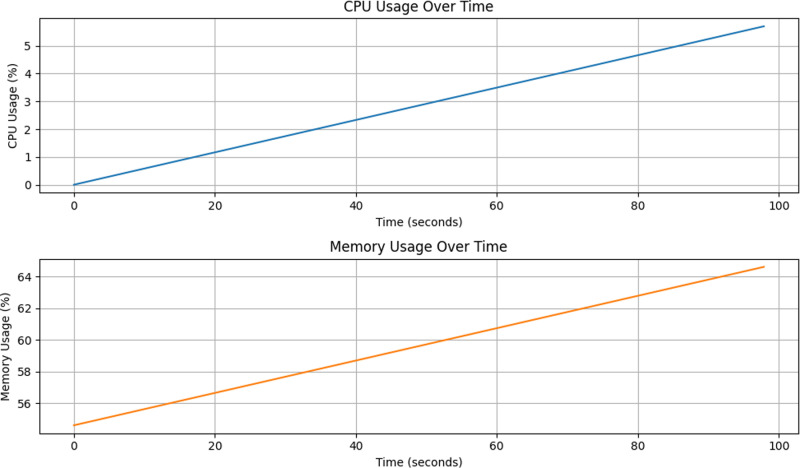
Dynamic threshold modeling performance overhead.

Fig 17 and Fig 18 demonstrate the change in computational overhead and model prediction performance after the introduction of the dynamic thresholding mechanism. As seen in Fig 17, although the dynamic thresholding mechanism increases the maximum CPU occupancy from 3% to 6% under the fixed threshold, this change does not have a significant impact on the stability and computational efficiency of the system. Fig 18, on the other hand, shows that the model prediction performance is significantly improved, indicating that the dynamic thresholding mechanism plays a key role in enhancing the model prediction capability. Further analysis shows that although the dynamic thresholding mechanism introduces a slight increase in computational overhead, by flexibly adjusting the thresholds, the model is able to more accurately capture the features of different categories, thus effectively improving the overall prediction performance. In addition, the memory occupancy rate is maintained at around 65% without significant fluctuations. In summary, despite the increase in computational overhead, the introduction of the dynamic thresholding mechanism significantly improves the prediction performance of the model, proving its effectiveness and feasibility in practical applications.

## 5 Conclusion

In our study, we apply an integrated approach of multiple classification models for server memory failure prediction. By conducting experiments on a large-scale dataset, our weighted voting integration approach combines the advantages of multiple models and significantly improves the prediction performance. In predicting server failures caused by DRAM errors, our integrated learning model achieves an F1 score of 74.1% and an accuracy of 84.5%, which is significantly better than a single classifier.

Despite some progress, current memory fault prediction models still have the following key issues: 1) insufficient diversity of base classifiers, mainly relying on traditional tree models, which are difficult to effectively capture complex fault patterns and temporal features; 2) feature engineering is too dependent on manual design, and fails to make full use of deep learning and other methods to automatically extract high-level features; and 3) the model structure is relatively simple, and it only fuses classifier outputs through voting fusing the outputs of classifiers in a way that fails to fully explore the synergistic relationship between classifiers. To address these issues, we propose a comprehensive improvement plan: first, to extend the expressive capability of the base classifiers, we will introduce LSTM models to capture long-term dependent features with memory errors, combine with the Transformer architecture to deal with heterogeneous data from multiple sources, while integrating rule-based expert systems to introduce domain knowledge, and systematically study the complementarities of different types of classifiers to optimize model Combination strategy. Second, in order to enhance the feature learning capability of the model, we will design an innovative second-level stacked integration architecture, including the construction of a feature importance assessment module to identify critical hardware configuration and load features, the introduction of an attention-based feature fusion layer to improve feature expression capability, the design of adaptive meta-classifiers to dynamically optimize the weights of the classifiers, and the implementation of synergistic optimization of the model components through the end-to-end training approach. Optimization. Finally, in order to improve the practicality of the system, we will focus on enhancing the online learning capability, including designing efficient incremental learning strategies to achieve continuous model evolution, developing a dynamic feature update mechanism to adapt to changes in the importance of features, constructing a real-time feedback mechanism for model evaluation, and investigating lightweight model updating strategies to achieve the optimal balance between performance improvement and computational overhead. Through these in-depth improvements, we expect to develop an in-memory CE-driven fault prediction system with enhanced robustness and adaptability, which not only significantly improves the prediction accuracy, but also demonstrates superior usability and maintainability in real-world application environments.
